# Emergency and disaster management training; knowledge and attitude of Yemeni health professionals- a cross-sectional study

**DOI:** 10.1186/s12873-018-0174-5

**Published:** 2018-08-06

**Authors:** Waheeb Nasr Naser, Huda Ba Saleem

**Affiliations:** 1 King Khalid Hospital Najran, King Abdul-Aziz Road, Najran, Kingdom of Saudi Arabia; 2 Faculty of Medicine and Health Sciences, Aden University, Aden, Yemen

**Keywords:** Training, Knowledge, Attitude, Emergencies, Disaster management, Health professionals, Yemen

## Abstract

**Background:**

Medical professionals together with other first responder teams are the first to attend an emergency or disaster. Knowledge and training in emergency and disaster preparedness are important in responding effectively.

This study aims to assess the current knowledge, attitude and training in emergency and disaster preparedness among Yemeni health professionals.

**Method:**

A descriptive, cross-sectional, non-probability based study was conducted in Yemen using self-reported on-line and paper surveys in 2017. A total of 531 health professionals responded. The Chi-Square test was used to identify any significant difference in the knowledge and attitude of the professional categories. The p-value of <0.05 was used as a statistical significant.

**Results:**

The overall knowledge status of Yemeni health professionals was insufficient with regards to emergency and disaster preparedness. Of all respondents, 32.0% had good knowledge, 53.5% had fair and 14.5% exhibited poor knowledge. The educational level was a key factor in the knowledge gap amongst respondents. Regardless of the period of experience, postgraduate staff were more knowledgeable than graduates. Physicians were better in knowledge than other subgroups of health specialties. Health administrators seemed insufficiently qualified in emergency and disaster planning. Medical teachers performed better in responding to knowledge test than managers. However, the majority of study respondents appeared in the ‘positive attitudes’ level to emergency and disaster preparedness. 41.0% of all respondents had received no courses in disaster preparedness. The trained staff used NGOs, and online-related programs more frequently for learning disaster planning (15.7%, and 13.6%) respectively. In contrast, formal resources such as MoPHP, health facility, medical schooling programs were used by (10.2%, 9.6, and 7.3%) of respondents, respectively. 58.9% of respondents had not participated in any exercise in emergency and disaster preparedness. Of all respondents, triage and mass causality response exercises were attended by only (13.5%, and 9.7%) respectively.

**Conclusion:**

The absence of teaching programs is a major issue in the lack of knowledge of health professionals regarding disaster preparedness. Thus, emergency and disaster preparedness has to be included in the primary medical school curricula and continuing medical education programs of the health facilities. Long-term formal training such as undergraduate and postgraduate programs is necessary. Operational simulations enrolled key personnel of multi-agencies focus on an organizational training rather than individual based training are recommended.

## Background

A disaster is a situation or event which overwhelms local capacity, necessitating a request to a national or international level for external assistance [1]. Global reports show that disasters are usually associated with a serious physical, mental, environmental and economic crisis to the affected vulnerable population [2–5]. Yemen is a disaster-prone country, with flooding being the main natural disaster [6]. Over the past several decades, due to poverty, social unrest, and civil conflicts, Yemen has experienced many human impact related emergencies. Recently, the huge complex humanitarian crisis caused by the ongoing war has left thousands killed or injured in this country [7].

Additionally, Yemen’s population has faced the world’s worst cholera outbreak [8, 9]. According to WHO, the country has been hit by two epidemic waves since October 2016 to the end of February 2018. The reported cumulative number of cholera cases is 1,097,735 including 2,392 related deaths with a case fatality rate of 0.22% [9]. In 2017, an Inter-Agency Standing Committee (IASC) in collaboration with the European Commission based on the physical exposure, vulnerability and the socio-economic status of the country ranked Yemen with a Risk Index 7.6 of 10. (INFORM) a measure of risk management to disaster and humanitarian crisis [10].

At the time of response to disasters and emergencies, the priority is to help, support and treat the victims; to save lives. Therefore, disaster relief and assistance are mainly carried out by rescue or emergency medical teams [11]. Moreover, when an emergency event such as fire occurs for instance; within the health facility, medical professionals will be on the frontline. Thus, they must be knowledgeable in disaster management and able to respond effectively to any disaster and emergency crisis. Education and training are necessary for health professionals to gain knowledge and develop the skills that make an effective response to disaster and emergency possible [12]. However, the lack of training programs in disaster preparedness was one of the main issues contributing to the negative outcomes of two regional studies on Yemen assessing hospital disaster preparedness [13, 14].

To the best of our knowledge, there has never been a national study carried out in Yemen evaluating the knowledge, attitude and training of health professional’s towards disaster management. Therefore, the main aim of this study is to assess the above issues.

Other specific objectives of the study are as follows:

1. To find out the training courses in emergency and disaster medicine attended by Yemeni health professionals.
2. To ascertain common hazards that might cause disasters or emergencies on the local and national level.
3. To highlight the necessary educational and training programs that might help policymakers to improve emergency and disaster management.

## Methods

### Study design

This is a descriptive and cross-sectional study carried out in May through June 2017 using a self-reported survey of both web and paper form questionnaires.

### Study setting

The setting of this study is the Republic of Yemen. Five main governorates were purposefully selected for an on-site visit to collect the data using a paper survey. These include the capital Sana’a, Aden, Hadramout, Shabwa and Lahj governorates. The authors assumed that if any training programs and more knowledgeable staff could be found they would be in the main cities; Sana’a and Aden, where most of the teaching institutions and university hospitals are sited. Hence, over 50% of papers were distributed in those two cities. The on-site visits targeted both governmental and private hospitals as well as ambulance rescue points.

In order to expand the sample, and bearing in mind time limitations, funding and safety concerns related to the ongoing war, authors used an online survey to collect additional data from other governorates where possible. Moreover, the circulated online survey helped to reach to a wide range of eligible participants from other non-visited facilities or agencies such as primary health care centres, health authorities and non-governmental organizations (NGOs).

### Study population

The targeted subjects were the health professionals who provide health care to the Yemeni people at the time of the study under the auspices of the Ministry of Public Health and Population (MoPHP) of Yemen. The enrolled subjects were categorized based on educational level, professional career, specialty, type of facility, the period of experience and workplace and used as independent variables.

### Sample

A non-probability purposive sampling technique was used in selecting the samples. The total number of the targeted population was assumed unknown. Therefore, we used the Daniel formula to estimate the minimum sample size for the study [15, 16]. Where (p) a value of expected proportion considered as 50%, (z) the confidence interval of 95%, (d) an error of deviation of 5 % and the calculated minimum sample size was 384.

$$ \mathrm{n}=\frac{{\mathrm{z}}^2\ \mathrm{p}\left(1-\mathrm{p}\right)}{{\mathrm{d}}^2} $$

### Study Tool

Two types of self-reported questionnaires were used; a paper survey and an online survey of google drive. Authors developed the tool after an extensive search of relevant literature of similar studies [17–22]. To our knowledge, disaster medicine and training are still not included in the programs of MoPHP of Yemen. Furthermore, studies reported that neither an incident command centre nor an emergency plan has yet been implemented in Yemen’s health facilities. [13, 14]. Therefore, the questionnaires were designed to outline the basic principles of disaster management stages such as mitigation, preparedness and response.

The questionnaire consists of 28 items of both structured and open-ended questions, and divides into six sections as follows; 1) an introduction: describes the purpose of the study and illustrates how to answer the questions. 2) Knowledge test: includes 11 items of correct and incorrect questions. 3) Attitude test: includes six items of 3 points Likert scale questions (agree, disagree and not sure). 4) Training and practice: consists of three items of multiple choice questions. 5) Anticipated disasters: consists of two items of multiple chooses questions. 6) Demographic data: consists of six categories: an educational level, professional career, specialty, facility, the period of experience and workplace. The majority of Yemeni staff use the Arabic language; hence, the tool was made both in English and Arabic to simplify an item's meanings. Furthermore, to test the tool validity before use, a pilot study was conducted on 50-health personnel. It was excluded from the main data. The authors used the feedback from the pilot study respondents to revise the questionnaire content.

### Data collection

Before the start, data collectors were trained in how to clarify respondent’s enquiries about the questions content. In addition to a list of emails and mobile numbers that were assembled from the contact list in some hospitals, the authors used WhatsApp, Facebook messenger, and groups to distribute the online survey to other professional colleagues and texted mobile messages to those not using these applications. The online survey link sent through email, mobile numbers and social media groups of the health personnel was left open for two months.

Within the web survey timeline, the paper survey was distributed to the health facilities of the selected cities as enumerated above. All non-medical profession and non-health administrative staff, as well as the piloting respondents, were excluded from this evaluation. Furthermore, 27 of the online survey respondents were found to be non-health professionals and were therefore removed from the study sample. A total of 531 eligible responses were gathered within the timeline of the study; 300 responses to the paper survey, and 231 to a web survey.

### Data analysis

The data collected from both survey types was compiled and then introduced into an Excel spreadsheet for coding. It was then transferred to the SPSS version 23 for analysis. Based on a similar study [19], the data was analyzed to get the final scores of the staff knowledge and attitude. In the knowledge test, the participants could get one score for each correctly answered question and zero for an incorrect answer. The maximum score was 11. Respondents with total correct scores of 9 or higher were graded “good,” those with scores between 5 and 8 were graded “fair” and those with scores of 4 or less were graded as having “poor” knowledge. In the attitude test; each question with an ‘agree’ response was scored 5 marks and both ‘disagree’ and ‘not sure’ were scored 0, with the maximum score of 30. The final respondent’s attitude was said to be positive if the respondent scored ≥15, and negative when a participant scored <15. Chi-square was used to compare the difference of knowledge and attitude status between the independent categorical variables. A p value of < 0.05 was used as a statistical significant cut off point. Other sections were analyzed by obtaining the frequencies and percentages.

### Ethical issues

An official written consent to carry out the study was obtained from the Research and Ethical Committee of the Faculty of Medicine and the Health Sciences University of Aden. To avoid having different approaches of informed consent because we have an online and in place participants, Research Committee agreed on having verbal consent. Therefore, during the visits, verbal permission to question the staff was granted by the facility’s administrators. Although the participation was voluntary, verbal consent was obtained from the respondents who were ensured the study was for educational purposes and the findings would be treated as facts.

## Results

### Demographic characteristic of the respondents

A total of 531 health professionals responded to the questionnaire. Of all respondents, 66.7% were graduates and 33.3% post-graduates. In respect to their position or career in the facility, around two-thirds were practitioners, 9.8% medical teachers and 16.0% managers. Of all respondents, only 6.4% were health administrators and the others were physicians and non-physician medical staff (mostly nurses) (50%, 44.6%) respectively. Around three-quarters of respondents were practicing in the public facilities and another was in private or non-profit facilities. One-half of all respondents had less than 5 years’ experience, while the remaining had from 5-10 years or more than 10 years (33%, 17.5%) respectively. Of all cities enrolled in the current study, 26.6% of respondents were in Aden, 25.2% Sana’a and the remaining were from other governorates (Table 1).

**Table 1:** The demographic characteristics of respondents

Variable	Frequency	Percentage
Educational level		
Post graduate	177	33.3
Graduate	354	66.7
Professional Career		
Medical teacher	52	9.8
Practitioner	394	74.2
Manager	85	16.0
Specialty		
Physician	260	49.0
Non-physician medical staff^a^	237	44.6
Health administrator	34	6.4
Facility		
Governmental facility	403	75.9
Non-governmental facility	128	24.1
Experience		
Less than 5 years	263	49.5
5-10 y	175	33.0
More than 10 y	93	17.5
Workplace		
Sana’a	134	25.2
Aden	141	26.6
Hadramout	60	11.3
Taiz	34	6.4
Lahj	41	7.7
Abyan	32	6.0
Shabwa	44	8.3
Other	45	8.5

^a^Non-physician medical staff such as nurses, paramedics, technicians, pharmacists

### Anticipated national and facility disasters

**Fig. 1:**
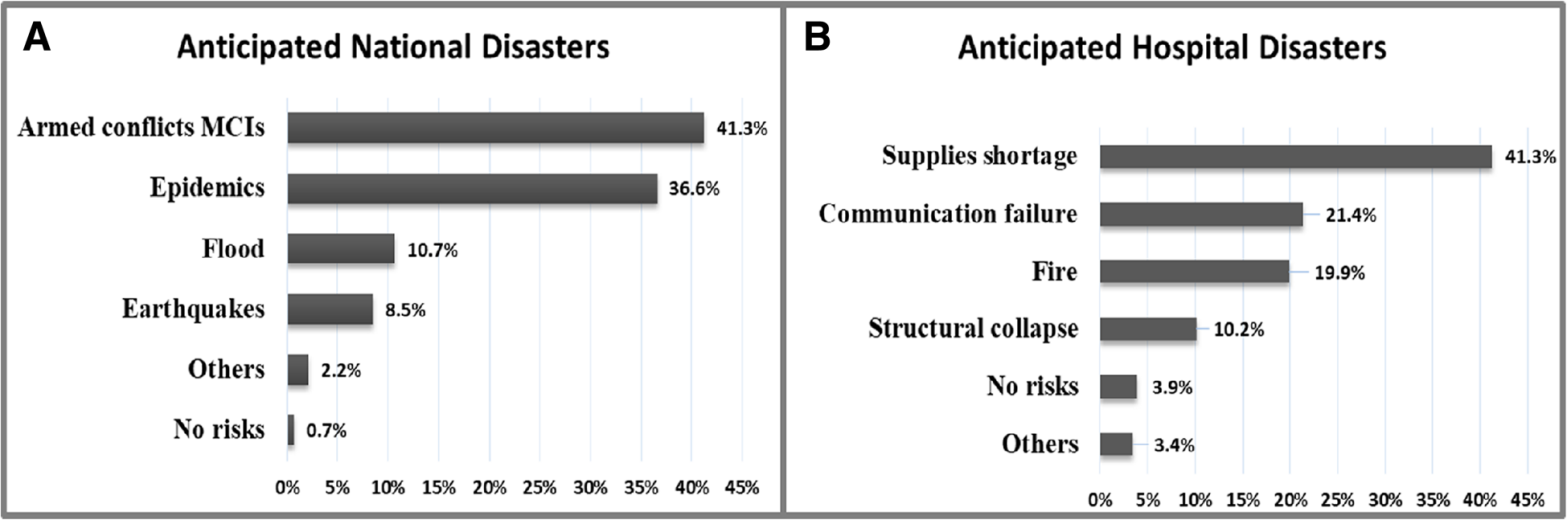
The frequency of emergencies or disasters expected by the respondents. **a** National or local emergencies or disasters. **b** Hospital emergencies or disasters

Violence due to either armed conflicts and/or terrorism was the major hazard concerning 41.3 % of subjects that might lead to mass casualty incidents, followed by pandemic, flood, earthquake (36.6%, 10.7, and 8.5%) respectively. While 2.2% of respondents were expecting other threats such as transportation accidents, drought and famine; 0.7% cited no risk Fig. 1a**.** A shortage of medical supplies is the main threat that might cause a hospital emergency crisis. It was ticked by 41.26% of respondents. Communication failure, fire, structural collapse was 21.4%, 19.9, and 10.2% respectively. 3.4% of respondents expected other potential hazards, such as chemical spillage or security threats, and 3.9% said no risks Fig. 1b.

### Training in disaster medicine

Media was the frequent informative source for disasters and emergencies in more than one-third of the study population (35.9%). The facility, schooling lessons, MoPHP and NGO related programs were indicated by 15.2%, 14.7, 14%, and 12.4% respectively. Self-reading was the source of information for 3.9% of participants. A negligible number had no source (3.5%).

**Fig. 2:**
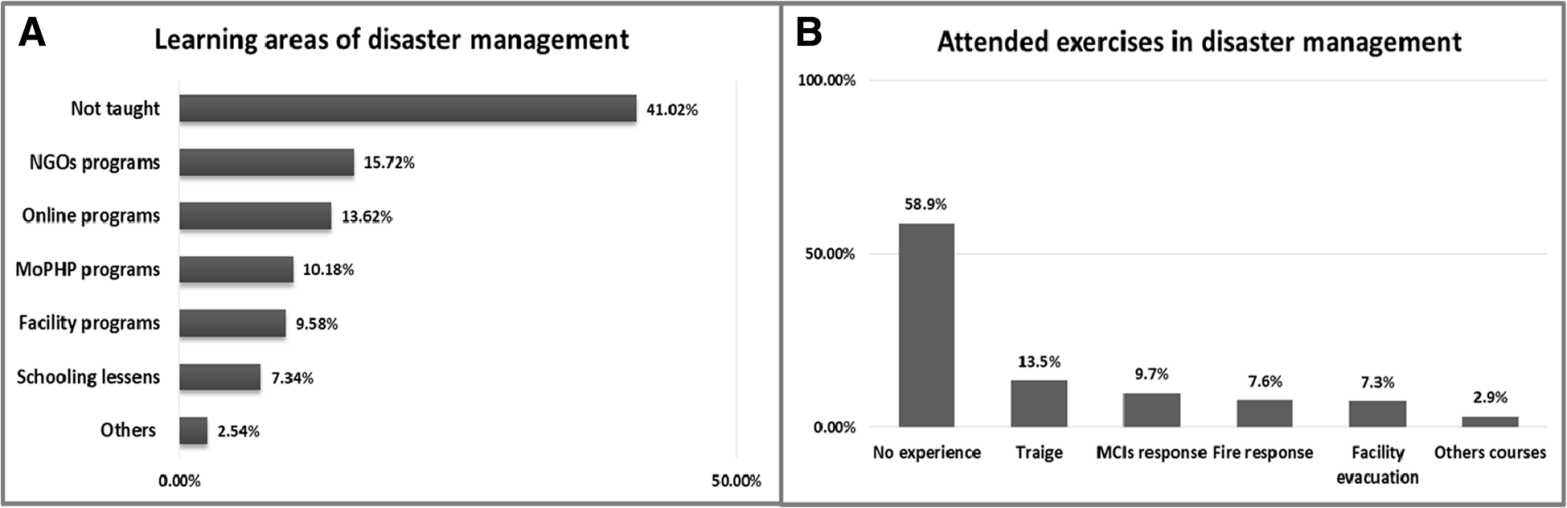
Emergency and disaster management. **a** The learning sources used by respondents. **b** The exercises or workshops attended by respondents

Of the study sample, 41.0% had not been taught disaster planning. Amongst those who had been taught, NGOs were the main learning source (15.7%) followed by online programs (13.6%). MoPHP, health facility related programs and schooling lessons accounted for 10.2%, 9.6, and 7.3% respectively. Additionally, 2.5% of participants used others ways of learning such as such self-study Fig. 2a. More than half of enrolled health professionals (58.9%) had not received any training courses in emergency and disaster preparedness. The remainder had received courses in triage, mass causality responses, fire responses and evacuation drills (13.5, 9.7, 7.6, 7.3%) respectively. Other courses such as first aid and infection control workshops were cited by 2.9% Fig. 2b.

### Disaster knowledge

**Fig. 3:**
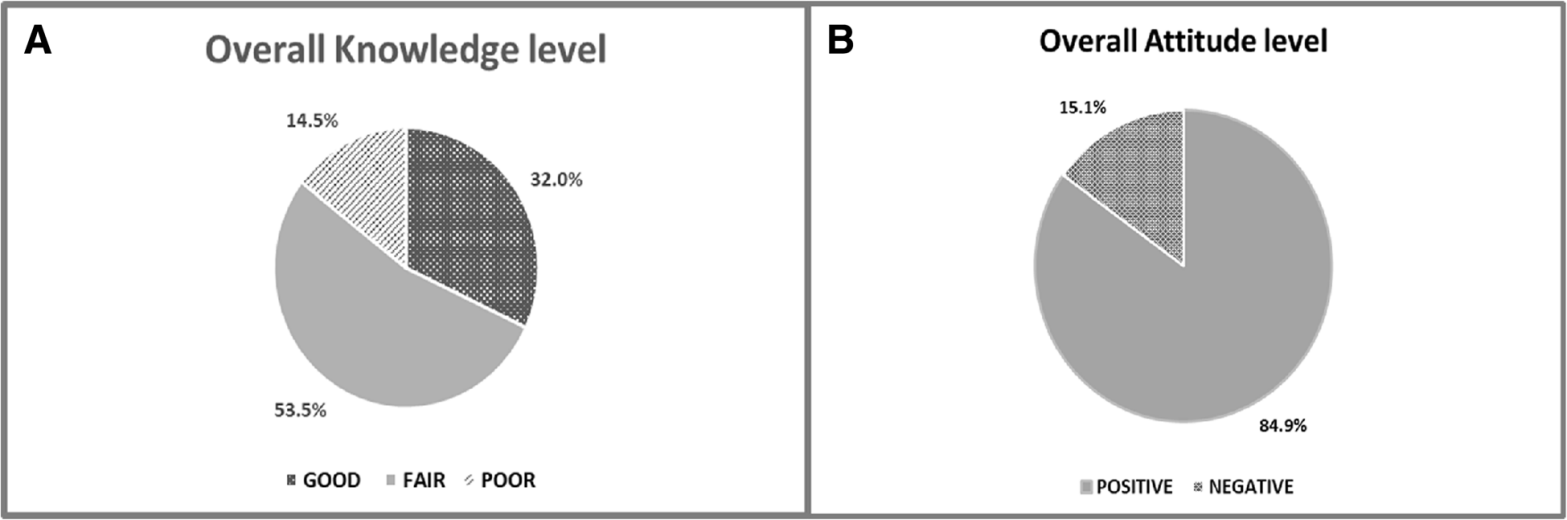
The overall scores of the respondents toward emergency and disaster preparedness; **a** Knowledge status of the respondents. **b** The attitude level of the respondents

Table 2 depicts the correct answer rates to the 11 knowledge questions (Q1-Q11). Overall, about one-third of participants (32.0%) had good knowledge, while the others had fair or poor knowledge in disaster management (53.5%, and 14.5%) respectively Fig. 3a. Moreover, there was a significant difference (*p* < 0.05) in knowledge level between graduates and postgraduates. Postgraduates responded better to almost all knowledge test questions. As far as professional experience was concerned, there was no significant difference in the individual’s knowledge level related to their level of experience Table 3.

**Table 2:** Knowledge and attitude of health professionals regarding emergency and disaster preparedness

Knowledge items	YES		NO
Have you heard about disaster?	501 (94.4)		30 (5.6)
Have you ever been taught about disaster planning?	256 (48.2)		275 (51.8)
Have ever performed a disaster drill(s) or workshop(s) in your facility or city?	166 (31.3)		365 (68.7)
A disaster is an imbalance between the demands that caused by an events and an available resources?	319 (60.1)		212 (39.9)
A disaster can occur either from natural or man-made causes?	377 (71.0)		154 (29.0)
Did you think, one day your country/city might be affected by disaster?	454 (85.5)		77 (14.5)
Did you think, one day your facility might be affected by disaster?	372 (70.1)		159 (29.9)
A disaster planning is to prepare to what might be needed to be done, how to be done, before and after disaster?	323 (60.8)		208 (39.2)
The surrounding hazards that most likely causing disaster to your facility most be identified and dealt with?	378 (71.2)		153 (28.8)
A disaster management it is includes both a health or non-health professional employees in the facility?	334 (62.9)		197 (37.1)
A disaster management it is an integral collaborative action of different agencies such as the hospitals, local health authority, civil defense and others?	347 (65.3)		184 (34.7)
Attitude items			
Training in disaster planning should be taught in your country	agree	disagree	No sure
Training in disaster planning is necessary in each health facilities.	451 (84.9)	24 (4.6)	56 (10.5)
It is necessary to have an emergency plan in your facility, city or country for any anticipated hazards.	438 (82.5)	26 (4.7)	67 (12.5)
It is necessary to have a disaster management committee in your facilities?	377 (71.0)	23 (4.3)	131 (24.7)
It is necessary to know your duty(s) and role(s) during disaster response in your facility.	340 (64.0)	35 (6.5)	156 (29.5)
To improve disaster management, is a training through the stimulation exercises, drills or workshops should be provided.	411 (77.4)	25 (4.7)	95 (17.9)

**Table 3:** Health professionals correctly answering the knowledge section test based on an educational and experience categories

		Educational level				Experience period			
Disaster knowledge related questions		Total	Graduate	Post graduate	*P* value	Less than 5 years	5 – 10 years	More than 10 years	*P* value
Q1	Background of disaster	501 (94.4)	331 (93.5)	170 (96.0)	0.232	249 (94.7)	167 (95.4)	85 (91.4)	0.376
Q2	Disaster planning background	256 (48.2)	140 (39.5)	116 (56.5)	< 0.001*	114 (43.3)	89 (50.9)	53 (57.0)	0.054
Q3	Disaster training background	166 (31.3)	92 (26.0)	74 (41.8)	< 0.001*	71 (27.0)	58 (33.5)	37 (39.8)	0.059
Q4	Disaster concept	319 (60.1)	201 (56.8)	118 (66.4)	0.028*	150 (57.0)	112 (64.0)	57 (61.3)	0.334
Q5	Sources of disaster	377 (70.1)	235 (66.4)	142 (80.2)	0.001*	176 (66.9)	134 (76.6)	67 (72.0)	0.090
Q6	Country vulnerability to disaster	454 (85.5)	287 (81.1)	167 (94.4)	< 0.001*	224 (85.2)	151 (86.3)	79 (84.9)	0.936
Q7	Facility vulnerability to disaster	372 (70.1)	233 (65.8)	139 (78.5)	0.003*	174 (66.2)	131 (74.9)	67 (72.0)	0.135
Q8	Concept of disaster planning	323 (60.8)	200 (56.5)	123 (69.5)	0.004*	151 (57.4)	113 (64.6)	59 (63.4)	0.275
Q9	Hazard vulnerability analysis	378 (71.2)	233 (65.8)	145 (81.9)	< 0.001*	169 (64.3)	130 (74.3)	79 (84.9)	< 0.001*
Q10	Response team	334 (62.9)	201 (56.8)	133 (75.1)	< 0.001*	155 (58.9)	117 (66.9)	62 (66.7)	0.173
Q11	Mutual collaboration	347 (65.3)	214 (60.4)	133 (75.1)	0.001*	160 (60.8)	127 (72.4)	60 (64.5)	0.040*

*Statistically significant

With regards to professional specialties, there was a significant difference in eight items of the knowledge test (*p* < 0.05). Physicians represented better than health administrators while non-physician medical staff did not get any higher score than other specialty subgroups. Regarding professional career, there was no significant difference in knowledge status amongst the career subgroups except the medical teachers responded correctly to Q2, Q3, Q9 with (*p* = < 0.05). The other questions had no significant difference between the career subgroups Table 4.

**Table 4:** Health professionals correctly answering the knowledge section test based on the specialty and professional career categories

		Specialties					Career			
Disaster knowledge related questions		Total	Physician	NPM staff$	Health Admin	*P* value	Medical Teacher	Practitioner	Managers	*P* value
Q1	Background of disaster	501 (94.4)	249 (95.8)	218 (92.0)	34 (100)	0.064	49 (94.2)	371 (94.2)	81 (95.3)	0.919
Q2	Background disaster planning	256 (48.2)	151 (58.1)	86 (36.3)	19 (55.9)	< 0.001*	35 (67.3)	175 (44.4)	46 (54.1)	0.004*
Q3	Background Disaster training	166 (31.3)	98 (37.7)	52 (21.9)	16 (47.1)	< 0.001*	22 (42.3)	112 (28.4)	32 (37.6)	0.049
Q4	Disaster concept	319 (60.1)	163 (62.7)	133 (56.1)	23 (67.6)	0.212	24 (46.2)	240 (60.9)	55 (64.7)	0.079
Q5	Sources of disaster	377 (71.0)	194 (74.6)	158 (66.7)	25 (73.5)	0.141	30 (57.7)	286 (72.6)	61 (71.8)	0.083
Q6	Country vulnerability to disaster	454 (85.5)	237 (91.1)	191 (80.6)	26 (76.4)	0.001*	47 (90.4)	334 (84.8)	73 (85.9)	0.555
Q7	Facility vulnerability to disaster	372 (70.1)	196 (75.4)	158 (66.7)	18 (52.9)	0.008*	43 (82.7)	272 (69.0)	57 (67.1)	0.104
Q8	Concept of disaster planning	323 (60.9)	177 (68.1)	122 (51.5)	24 (70.6)	< 0.001*	37 (71.2)	241 (61.2)	45 (52.9)	0.102
Q9	Hazard vulnerability analysis	378 (71.2)	199 (76.5)	153 (64.6)	26 (76.5)	0.010*	45 (86.5)	266 (67.5)	67 (78.8)	0.004*
Q10	Response team	334 (62.9)	185 (71.2)	126 (53.2)	23 (67.6)	< 0.001*	39 (75.0)	242 (61.4)	53 (62.4)	0.162
Q11	Mutual collaboration	345 (65.0)	185 (71.2)	141 (59.5)	21 (61.8)	0.022*	40 (76.9)	253 (64.2)	54 (63.5)	0.180

*Statistically significant, $ Non-physician medical staff

### Attitude to disaster

Table 2 shows the agreed upon responses of study participants to the attitudes test (Q12-Q17). The study concluded that the respondent’s attitude toward disaster management was generally positive. Of study participants, 84.9% agreed to the teaching of disaster management in the country (Q12). They want to have an emergency plan, to know their roles during the response to emergency events, as well as wanting to train on disaster planning Fig. 3b.

The findings also elicited that the level of attitude regarding disaster planning among the health professions was statistically different. Postgraduates agreed with most attitude questions compared to graduates (*p* < 0.05). The length of experience seemed to have no effect on the professional attitude towards disaster management. However, those who had worked a long time appeared slightly more eager for training and the implementation of an emergency plan in their workplaces Table 5.

**Table 5:** Health professionals agreed to an attitude section test based on their educational and experience level categories

		Educational level				Experience period			
Disaster management attitude related questions		Total	Graduate	Post graduate	*P* value	Less than 5 years	5 – 10 years	More than 10 years	*P* value
Q12	Disaster training in country.	451 (84.9)	295 (83.4)	156 (88.1)	0.145	218 (82.9)	148 (84.6)	85 (91.4)	0.141
Q13	Disaster training in facility	438 (82.5)	284 (80.3)	154 (87.0)	0.053	211 (80.2)	149 (85.1)	78 (83.9)	0.385
Q14	Availability of emergency plan	377 (71.0)	241 (68.1)	136 (76.8)	0.036*	184 (70.0)	118 (67.4	75 (80.6)	0.066
Q15	Disaster management committee	340 (64.0)	209 (59.0)	131 (74.0)	0.001*	161 (61.2)	118 (67.4)	61 (65.6)	0.391
Q16	Roles and responsibility	411 (77.4)	265 (74.9)	146 (82.5)	0.048*	197 (74.9)	144 (82.3)	70 (75.3)	0.168
Q17	Hand on exercises vs lectures in disaster training	440 (82.9)	279 (78.8)	161 (90.9)	< 0.001*	203 (77.2)	152 (86.9)	85 (91.4)	0.002*

*statistically significant

Health administrators were interested in implementing simulation-training programs in their facilities. They considered that hands-on or field exercises and workshops were an appropriate method in disaster training rather than lectures and presentations (*p*< 0.05). Physicians preferred to know their role during disaster and emergency responses in their workplaces (*p* < 0.05). Among the careers, medical teachers appeared higher in attitude to disaster management, in terms of risk analysis, planning and committee supervision (*p* < 0.05). Leaders or managers were higher in attitude to hospital and field hands-on training Table 6.

**Table 6:** Health professionals agreed to an attitude section test based on the specialty and professional career categories

			Specialties				Career			
Disaster management attitude related questions		Total	Physician	NPM staff$	Health Admin	*P* value	Medical Teacher	Practitioner	Managers	*P* value
Q12	Disaster training in country.	451 (84.8)	223 (85.8)	196 (82.7)	32 (94.1)	0.191	47 (90.4)	335 (85.0)	69 (81.1)	0.342
Q13	Disaster training in facility	438 (82.5)	221 (85.0)	185 (78.1)	32 (94.1)	0.023*	44 (84.6)	322 (81.7)	72 (84.7)	0.773
Q14	Emergency plan	377 (71.0)	190 (73.1)	166 (70.0)	21 (61.8)	0.357	43 (82.7)	268 (68.0)	66 (77.6)	0.031*
Q15	Disaster management committee	340 (64.0)	178 (68.5)	142 (59.9)	20 (58.8)	0.113	41 (78.8)	240 (60.9)	59 (69.4)	0.021*
Q16	Roles and responsibility	441 (77.4)	212 (81.5)	180 (75.9)	19 (55.9)	0.003*	41 (78.8)	308 (78.2)	62 (72.9)	0.559
Q17	Hand on exercises vs lectures in disaster training	440 (82.9)	232 (89.2)	177 (74.7)	31 (91.2)	< 0.001*	47 (90.1)	316 (80.2)	77 (90.5)	0.022*

*statistically significant, $ Non-physician medical staff

## Discussion

The present study aimed to ascertain the current knowledge, attitude and training attributes among Yemeni health professionals in relation to disaster management. The findings indicate that the overall knowledge level of the health professionals was insufficient and needed improvement. Only 32% of health professionals were knowledgeable about disaster management, with 53.5% and 14.5% expressing fair and poor knowledge respectively Fig. 3a.

This unsatisfactory outcome status was reported in some studies conducted worldwide for the same purpose [19, 20, 22–25]. For instance, studies conducted via a non-probability sampling method on selected tertiary hospitals in Lagos, Nigeria [19] and Nairobi, Kenya [20]. Healthcare and non-healthcare professionals in the hospitals were evaluated and the overall level of knowledge among the staff was 47.8% and 36% in Lagos and Nairobi respectively. A study in Shanghai, China [22] used a probability sampling for medical professionals and medical students and random sampling for community residents. Two different tools were conducted and it concluded that health professionals were more knowledgeable than medical students. Community residents displayed very poor knowledge. However, educational level showed statistical significance in their knowledge level towards disaster management. In Madinah, KSA [24], a study carried out on two batches of postgraduate nursing students with at least ten years prior working experience. The sample was obtained via a non-probability method and the findings revealed that the knowledge and training levels were below acceptable levels.

In contrast to the present results, international studies showed that staff had good enough knowledge regarding disaster management [25, 26]. Pre World Cup South Africa in 2010, a study conducted in a teaching tertiary hospital in Johannesburg [25] showed an overall acceptable knowledge base. Although the results were acceptable, it was recommended that there was room for improvement among the training staff. This came up despite the fact that staff undergo regular training courses in disaster management and the hospital had a disaster committee with an already implemented action plan for emergencies. In Mecca, Saudi Arabia, a non-probability based study targeted all registered nurses working in the emergency departments of all four public hospitals in Mecca [26]. Results showed that most emergency nurses appeared to be confident and knowledgeable about their roles in responding effectively to mass gathering disasters. However, their knowledge of other disaster types was still insufficient despite their frequent training in the hospitals. Thus, both hospital and university-based training programs were recommended.

In the current study, a significant difference in knowledge level was exhibited between postgraduates and graduates regardless of the length of experience. More than 90 % of responses to knowledge items presented were statistically different in relation to the educational level of the respondents. In contrary, more than 90 % of responses to knowledge test were not statistically different, based on the length of experience (*P* < 0.05) Table 3. It seems that graduated participants had limited exposure to training programs. One possible explanation for this is the lack of disaster training programs in medical schools and during the continuing medical education (CME) programs in the health facilities. On the other hand, findings showed that some programs were conducted for postgraduates. Nonetheless, the study did not specify whether the postgraduate programs were held within or outside the country. Therefore, this is still an issue that needs to be particularly addressed and further study is suggested.

Health administrators even with the good background to disaster and emergencies as reflected in Q1 and Q3, did not meet their expected proficiency in disaster management. They underestimated the risk analysis, the importance of the mutual aids and multidisciplinary collaboration role in disaster planning. Therefore, training programs in leadership skills might enhance the provision of health administrator’s capacity with respect to disaster. Physicians enjoyed higher knowledge level than other specialties. In agreement with these findings, there was a study which reported a similar outcome [22]. Managers also displayed insufficient knowledge in disaster management especially in the related items (Q7, Q9, Q10, and Q11). Meanwhile, medical teachers in general, appeared knowledgeable when compared to the managers and practitioners. However, they appeared to have a misunderstanding of disaster concepts and origins (Q4, Q5) and this lag could be attributed to the lack of disaster medicine faculties and/or instructors.

We can argue that the main reason for a shortcoming in Yemeni health professional’s knowledge regarding disaster management is the paucity of the formal training programs or lack of disaster medicine from the medical school curriculum. It explored merely some short-term courses focusing on specific disaster responses. Furthermore, 35.9% of the study subjects had heard about disaster from the media and only 14.7% through undergraduate school lessons. Moreover, only 41% of respondents had ever been trained in disaster medicine. Of those who had received training, a higher percentage 15.7% attended courses through NGOs and 13.6% through online programs compared to the negative role of formal institutions and facilities.

The present study revealed a positive attitude of respondents and exhibited their readiness to learn disaster management and desire to be prepared. These findings were reported in some international published studies [19, 21–25]. The respondents felt that their facilities should have an emergency plan with a disaster committee supervising the implementation of this plan. Furthermore, they should know their assignments when the plan has activated. The current study revealed a higher percentage of respondents agreeing that training is necessary for the country and in their facilities (84.8%, 82.5%) respectively. Moreover, 82.9 % confirmed that drills, workshops, or other simulations exercises must be conducted in their workplaces and they are perceived as being appropriate for disaster training.

In 1997, the Yemen government authorized the Supreme Council of Civil Defense (SCCD) of the Ministry of Interior to lead the disaster management in the country [27]. SCCD focused mainly on reactive responses and post-disaster relief [28]. In 2006, after a national flood disaster SCCD developed a national plan for emergencies and disasters, but it does not meet the needs. So there exists, a requirement for a national authority which will revise legislation and policies in regards to disaster management. The national authority should lead and oversee an emergency and disaster preparedness including training in the country.

Indeed, education and training are key elements of disaster preparedness [29]. Thus, for the strengthening of health professional’s ability in regards to an emergency and disaster management, provision of formal educational programs is necessary. Long-term training programs that have a comprehensive curriculum are more standardized than short courses [30]. Government has to establish undergraduate or postgraduate degree-based courses on disaster medicine either inside the country or overseas on scholarships. Blended learning programs, a mix of online lectures and classroom discussions followed by hands-on field workshops, drills or large-scale exercises are suggested. Operational-based exercises incorporating the key personnel of multi-agencies, which focus on leadership skills, team collaboration, communication and resources allocation decision-making rather than individual based performance, are necessary.

Additionally, based on the study findings, traumatic MCIs are frequent events that could cause a burden to healthcare providers, particularly surgical teams. Therefore, field first responders have to be trained in incident command system (ICS) and mass causality triage [31]. Other courses such as basic and advanced disaster life support (BDLS, ADLS) and prehospital trauma life support (PHTLS), advanced trauma life support (ATLS) are to be considered.

The government has to enforce disaster-planning training as a part of the orientation and CME programs of each health facilities. Stakeholders have to establish an emergency HICS, and plan for any disaster that could happen. Furthermore, the staff must be trained regularly to enhance their performance in order to respond effectively to such disasters [32]. The community also has to be integrated to disaster management. Public awareness of disaster risks, effects, and response when disaster strikes are key to a community surviving [33]. Moreover, to support the formal response efforts in disaster response, the government has to launch training programs for public volunteers. For that, search, rescue, evacuation and basic first aids skills courses are suggested. Aftermath disaster, mental support of the stressed victims is important [4]. Thus, psychological first aid skills courses have to be considered. Finally, study findings reported that infectious casualties found a common issue facing healthcare providers in their workplaces; hence, providing training programs in infection control measures, personnel protective equipment (PPE), surveillance, early warning and case-tracking systems are a must.

## Conclusion

A considerable number of Yemeni health professionals presented as ‘unknowledgeable’, with limited opportunities for training despite their beliefs towards disaster management. There was a gross lack of formal teaching and training programs in emergency and disaster medicine. Therefore, disaster-training programs are urgently needed, with specific emphasis on key personnel such as health administrators, facility managers, medical teachers, first responders and public health providers. It is recommended that disaster medicine be augmented either in the curriculum of undergraduate medical schools or in postgraduate university-based programs as well as in the continuing medical educations CME programs of MoPHP and health facilities of Yemen.

### Limitations

Several limitations were met in the current study. One limitation was the interest of the community to such research and internet availability. These issues have been addressed; authors used both online and paper forms to get a large enough sample for the study. Another issue was the size and technique of sampling used since there was no available data to get comparable proportional figures of health professionals, the limited time, funding and unsafe access to cities due to the ongoing war. Thus, researchers used the non-probability purposive sampling, using both an online and paper surveys to collect a large amount of data and to generalize the survey to the unvisited provinces to minimize the selective bias as much as possible.

The generalizability of findings has taken in the author’s concern. For the same reasons, all health professionals and provinces in Yemen will not get an equal chance to participate. Authors focused on the main urban cities i.e. Sana’a and Aden in addition to the feasible cities. Therefore, we can say the overall findings can reflect the current knowledge of the country. The origin of training among the taught respondents; was not specified in the questionnaire's items as to whether the training was held inside or outside the country. Thereby, it does not reflect the provided actual size of formal programs. Finally, the items’ content of the questionnaire. Since there was no emergency plan applicable in all health facilities to be tested in this survey, the authors just included the basic principles in disaster management in the questionnaire.

## Abbreviations

CME: Continuing Medical Educational
HICS: Hospital Incident Command System
ICS: Incident Command System
MCIs: Mass Causality Incidents
MoPHP: Ministry of Public Health and Population
NOGs: Non-Governmental Organizations
PHC: Primary Healthcare Centers
SCCD: Supreme Council of Civil Defense
WHO: World Health Organization
